# Importance of *H*-Abstraction in the Final Step of Nitrosoalkane Formation in the Mechanism-Based Inactivation of Cytochrome P450 by Amine-Containing Drugs

**DOI:** 10.3390/ijms141224692

**Published:** 2013-12-18

**Authors:** Hajime Hirao, Nandun M. Thellamurege, Pratanphorn Chuanprasit, Kai Xu

**Affiliations:** Division of Chemistry and Biological Chemistry, School of Physical and Mathematical Sciences, Nanyang Technological University, 21 Nanyang Link 637371, Singapore; E-Mails: nandun.t@ntu.edu.sg, (N.M.T.); pratanph001@e.ntu.edu.sg (P.C.); xuka0004@e.ntu.edu.sg (K.X.)

**Keywords:** cytochrome P450, mechanism-based inactivation, reaction mechanism, metabolic-intermediate complex, density functional theory, energy decomposition analysis

## Abstract

The metabolism of amine-containing drugs by cytochrome P450 enzymes (P450s) is prone to form a nitrosoalkane metabolic intermediate (MI), which subsequently coordinates to the heme iron of a P450, to produce a metabolic-intermediate complex (MIC). This type of P450 inhibition, referred to as mechanism-based inactivation (MBI), presents a serious concern in drug discovery processes. We applied density functional theory (DFT) to the reaction between *N*-methylhydroxylamine (NMH) and the compound I reactive species of P450, in an effort to elucidate the mechanism of the putative final step of the MI formation in the alkylamine metabolism. Our DFT calculations show that *H*-abstraction from the hydroxyl group of NMH is the most favorable pathway via which the nitrosoalkane intermediate is produced spontaneously. *H*-abstraction from the N–H bond was slightly less favorable. In contrast, *N*-oxidation and *H*-abstraction from the C–H bond of the methyl group had much higher energy barriers. Hence, if the conversion of NMH to nitrosoalkane is catalyzed by a P450, the reaction should proceed preferentially via *H*-abstraction, either from the O–H bond or from the N–H bond. Our theoretical analysis of the interaction between the MI and pentacoordinate heme moieties provided further insights into the coordination bond in the MIC.

## 1. Introduction

Human cytochrome P450 enzymes (P450s) are known as versatile biological catalysts with remarkably broad substrate specificity [1–14]. A variety of different drugs are metabolized by only a few P450 isozymes, mainly by CYP2B6, CYP2C9, CYP2C19, CYP2D6, and CYP3A4 [15]. Malfunction of these P450s through drug–drug interaction (DDI) is causally linked to unfavorably altered metabolic profiles of compounds. *In silico* methods hold significant promise for predicting and minimizing the risks of DDIs at an early stage of a drug discovery project. However, *in silico* description of a particular type of DDI, referred to as mechanism-based inactivation (MBI) [16–23], presents a difficult challenge because MBI occurs via P450-catalyzed metabolic intermediate (MI) formation. To describe such reactive processes computationally, one must resort to quantum chemistry. In recent years, quantum chemical studies of P450 MBI using density functional theory (DFT) have become increasingly prevalent [24–29].

**Scheme 1.**
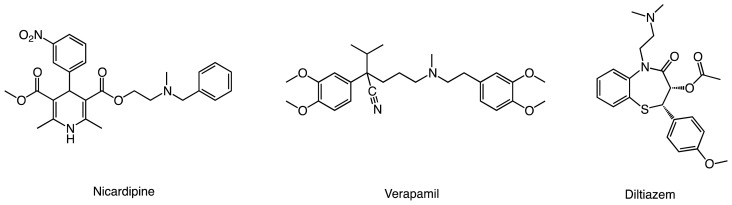
Examples of amine-containing calcium channel blockers that act as mechanism-based inactivators.

**Scheme 2.**
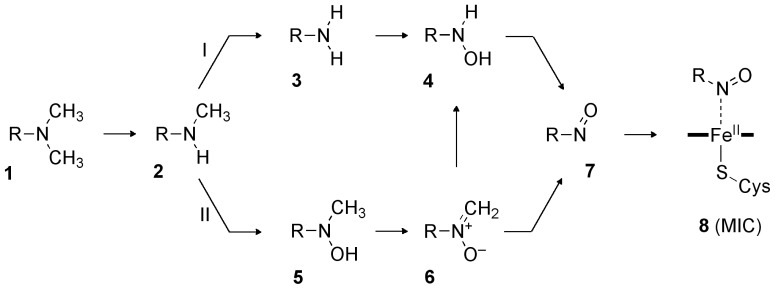
Possible pathways of metabolic-intermediate complex (MIC) formation starting from a tertiary amine [38]. Some of the alkyl groups are replaced by CH_3_ for simplicity.

Of all compounds that inhibit P450s through DDI or MBI, alkylamines are a particularly important class because they include a number of drugs such as calcium channel blockers (Scheme 1), macrolide antibiotics, monoamine oxidase inhibitors, *etc*. [30–34] Tertiary, secondary, and primary alkylamines have been reported to cause quasi-irreversible-type MBI via formation of a nitrosoalkane MI. As illustrated in Scheme 2, a tertiary alkylamine (**1**) is dealkylated to a secondary alkylamine (**2**) [35–37], which means that tertiary and secondary alkylamines follow a common pathway for the formation of a MI (**7**). The metabolism of secondary alkylamine is somewhat controversial, because **2** may follow either path I or II [38]. However, it is often believed that a *N*-hydroxyalkylamine intermediate (**4**) is formed in either case, just before the MI formation [19,22,23,39,40]. The conversion of **4** to **7** may therefore be regarded as a critical step that all alkylamines pass through, before forming a MI. The resultant MI coordinates to the ferrous heme iron to form a MI complex (MIC, **8**), which is directly responsible for the enzyme inhibition and features a Soret absorbance peak at ~455 nm [41].

This study is particularly concerned with the putative final step of the nitrosoalkane MI formation, *i.e.*, conversion of **4** to **7** and the coordination of **7** to the heme. We examine the mechanism in which an oxoiron(IV) porphyrin π-cation radical intermediate, compound I (Cpd I), of a P450, is responsible for this oxidative process, although it should be mentioned that **4** may not always require a P450 for the oxidation because it readily undergoes autoxidation [39,42]. As this step is not well explored, we here attempt to find a plausible reaction mechanism using density functional theory (DFT) calculations. Moreover, we investigate the nature of the coordination bonds in ferrous and ferric MICs.

## 2. Results and Discussion

### 2.1. Models

**Scheme 3.**
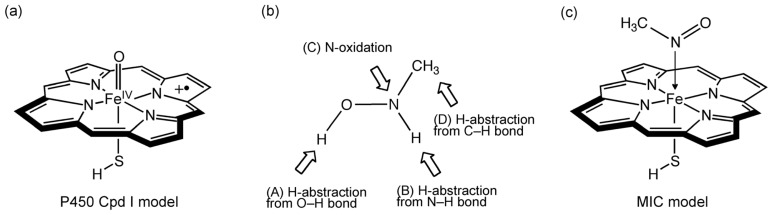
(**a**) Cpd I model; (**b**) Hydroxylamine and three possible pathways considered (paths A–D); and (**c**) MIC model.

For the calculations, we used a Cpd I model as depicted in Scheme 3a. The R group of *N*-alkylhydroxylamine (**4**) in Scheme 2 was assumed to be CH_3_; that is, *N*-methylhydroxylamine (NMH) was used (Scheme 3b). As shown in Scheme 3b, four possible pathways A–D for the reactions between Cpd I and **4** were considered, which respectively begin with *H*-abstraction from the O–H bond (path A), *H*-abstraction from the N–H bond (path B), *N*-oxidation (path C), and *H*-abstraction from the methyl group (path D). It did not seem plausible that electron transfer occurs from the substrate to Cpd I prior to bond formation (Scheme S1).

### 2.2. Reaction Mechanism

**Figure 1.**
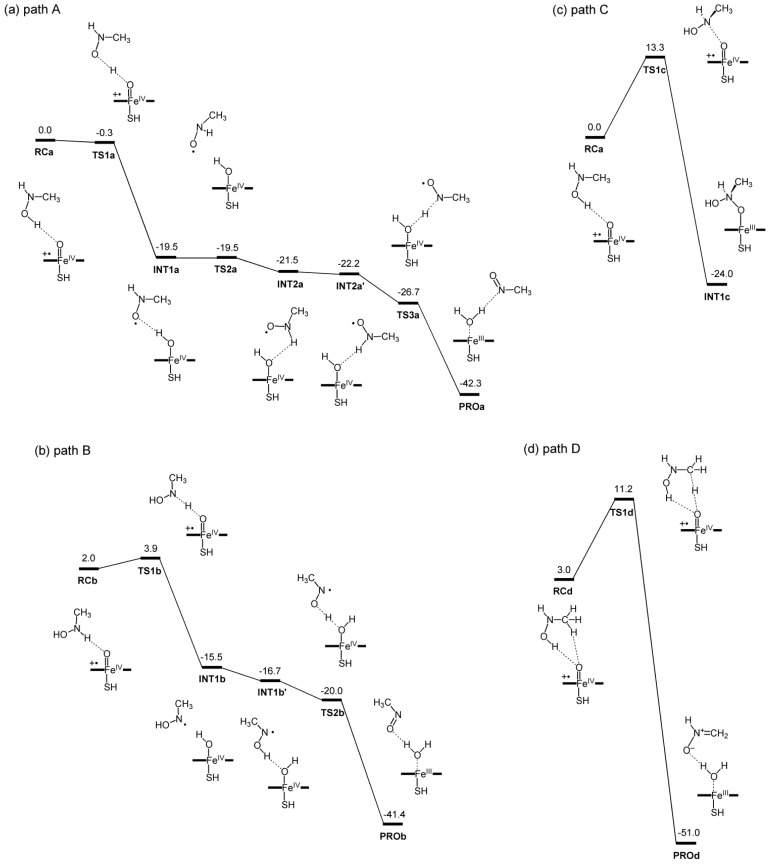
Energy diagrams (in kcal/mol) for (**a**) path A; (**b**) path B; (**c**) path C; and (**d**) path D, obtained at the B3LYP(SCRF)/B2//M06/B1 level with zero-point energy corrections.

**Figure 2.**
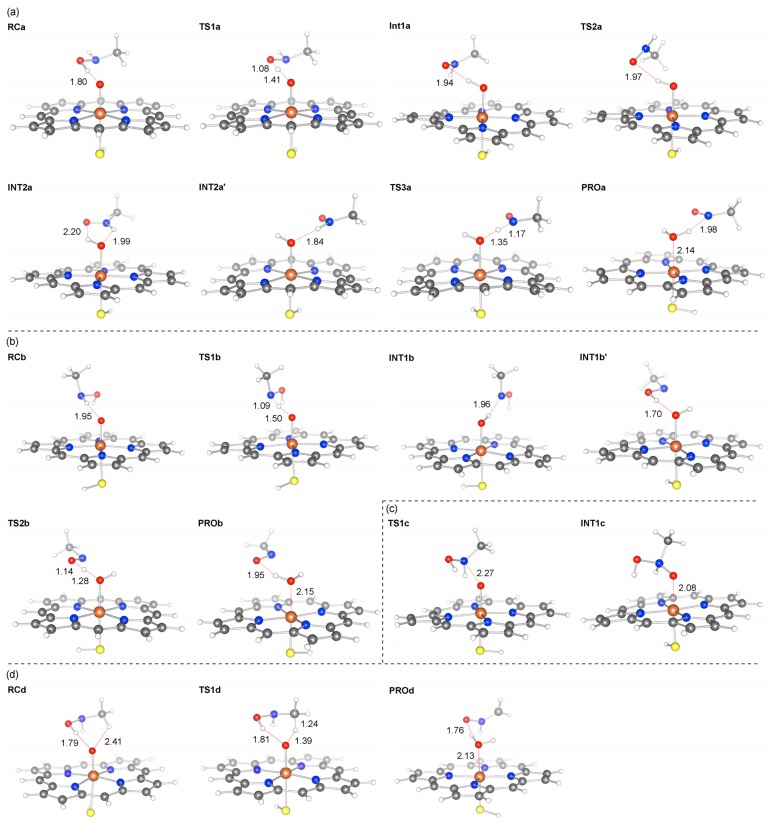
Optimized geometries of species on (**a**) path A; (**b**) path B; (**c**) path C; and (**d**) path D. Key bond distances are shown in Å.

The energy profiles for all four pathways are presented in Figure 1, and the optimized intermediates and transition states are shown in Figure 2. Raw energy data, group spin populations, and group atomic charges for all species are summarized in Tables S1–S5. The XYZ coordinates of optimized geometries are also available in the Supporting Information.

The energy profile and key geometries for path A are presented in Figures 1a and 2a, respectively. The reactant complex on this path (**RCa**) is stabilized by a hydrogen bond (H bond) between the hydroxyl group of NMH and the oxo moiety of Cpd I. The first *H*-abstraction occurs through a transition state, **TS1a. TS1a** is lower in energy by 0.3 kcal/mol than **RCa**, indicating that the *H*-abstraction step is barrierless. The *H*-abstraction leads to an intermediate, **INT1a**, which is a weakly interacting complex of ferryl-type Cpd II and a substrate radical. Subsequently, another hydrogen atom is abstracted from the N–H bond to form a product complex, **PROa**. There is no noticeable barrier in the second *H*-abstraction.

As seen in Figures 1b and 2b, path B begins by forming a reactant complex, **RCb**, which is stabilized by a H bond between the N–H bond of NMH and Cpd I. **RCb** is less stable than **RCa** by only a few kcal/mol, and the first *H*-abstraction from the N–H bond via **TSb** has a small energy barrier of ~2 kcal/mol. The *H*-abstraction leads to an intermediate, **INT1b**, which is a complex of Cpd II and a substrate radical. The second *H*-abstraction from the O–H bond en route to **PROb** has no barrier. Thus, the energy diagrams for the two *H*-abstraction pathways (paths A and B) in Figure 1a,b suggest that the MI formation from **4** through these pathways should be remarkably facile.

In contrast, the energy barrier (13.3 kcal/mol) existing on the *N*-oxidation pathway (path C, see Figure 1c) is much higher than those for paths A and B. Furthermore, a recent DFT study done by Taxak *et al*. [26] showed that the *N*-oxidation intermediate, **INT1c**, is subsequently converted to a *N*,*N*-dihydroxy-type diol intermediate, and that the dehydration of the diol has a very high energy barrier (~35 kcal/mol). It therefore seems reasonable to conclude that the reaction does not choose path C over path A or B for the MIC formation.

The energy barrier for *H*-abstraction from the methyl group (11.2 kcal/mol) on path D is somewhat lower than that for path C (Figure 1d). The *H*-abstraction from a C–H bond is followed by another spontaneous *H*-abstraction from the O–H bond, resulting in the formation of formaldonitrone at **PROd**. However, the barrier for path D is still much higher than those for paths A and B; thus, it is less likely that the reaction follows path D. Interestingly, the bond dissociation energies of the O–H, N–H, and C–H bonds correlated well with the calculated barrier heights for *H*-abstraction from these bonds (Table S6).

Taken together, our calculations suggest that species **4** is converted to **7** via path A or B.

### 2.3. Coordination Bond in MIC

**Figure 3.**
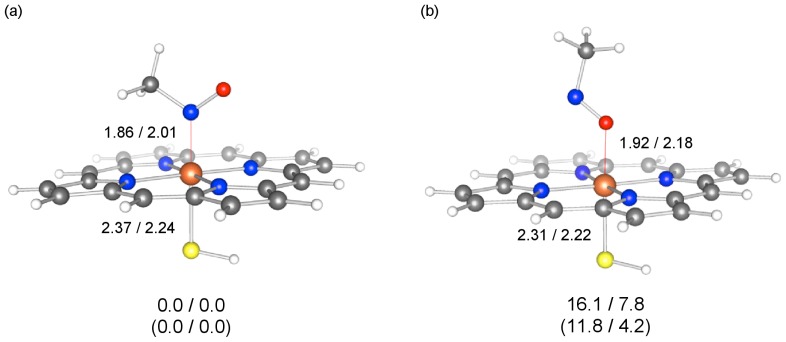
M06/B1-optimized geometries of ^1^MIC(II) and ^2^MIC(III): (**a**) the N-bound form and (**b**) the O-bound form. Key distances are given in Å. The values below the geometries are relative energies (kcal/mol) obtained at the M06(SCRF)/B2 level (^1^MIC(II)/^2^MIC(III)), while the values in parentheses are relative energies obtained at the B3LYP(SCRF)/B2 level.

The produced nitrosomethane species will coordinate to the heme iron to form a MIC. We investigated the nature of the coordination bond in the MIC, considering two different types of MICs, MIC(II) and MIC(III), in which the central iron has a formal oxidation state of +2 and +3, respectively. The spin states of these complexes were assumed to be singlet and doublet (*i.e.*, ^1^MIC(II) and ^2^MIC(III), where the superscripts stand for the spin multiplicity). For each of these MICs, we optimized the geometries of N-bound and O-bound forms, which respectively use the N and the O atom of the nitrosomethane for the coordination to Fe. Figure 3a,b show the optimized geometries of the N-bound and O-bound forms, respectively, along with their relative energies. A comparison of the energies of these two forms clearly shows that the N-bound form is more stable in both MIC(II) (by >11 kcal/mol) and MIC(III) (by >4 kcal/mol), which is in accordance with the conventionally assumed structure (Scheme 2) and the X-ray structures of related complexes and enzymes [43,44]. Interestingly, in Figure 3a, the Fe–N distance in the N-bound geometry is seen to be shorter for MIC(II) than for MIC(III). This trend implies that the interaction may be somewhat stronger in the ferrous MIC.

To evaluate the binding strengths of these MICs more quantitatively, we calculated the interaction energies (Δ*E*), or the energy change for the following processes:

(1) [FeII(Por)(SH)]5+nitrosomethane→M1IC(II)

(2) [FeIII(Por)(SH)]6+nitrosomethane→M2IC(III)

where Por and SH denote the porphine and SH^−^ ligands, respectively. In other words, we evaluated how the potential energy changes, when a pentacoordinate heme in a high-spin ground state binds to nitrosomethane to form a MIC [11]. Table 1 summarizes the calculated Δ*E* data. Three methods were examined, *i.e.*, M06, B3LYP, and B3LYP-D3. The calculations with the M06 functional predicted that the complex formation in MIC(II) and MIC(III) is a stabilizing and a destabilizing process, respectively, whereas B3LYP and B3LYP-D3 predicted that both interactions are favorable. Despite some differences in the magnitudes of Δ*E*, all methods predicted that the MIC formation is more favorable for the ferrous (Fe(II)) heme, which is consistent with the experimental observation that the iron in a nitrosoalkane MIC has a ferrous state [40].

### 2.4. Energy Decomposition Analysis of MIC

The nature of coordination bonds was further investigated using energy decomposition analysis (EDA). Table 2 summarizes the decomposed energy terms for the interaction between nitrosomethane and the ferrous or ferric heme group in the MIC (see Scheme 3 and Figure 3a). It should be noted that the total “interaction energy” evaluated here is somewhat different from those obtained from Equations (1) and (2) (Table 1) in that (i) the geometries of the fragments are the same as those in the MICs, and (ii) the [Fe(Por)(SH)] fragments are in low-spin states. Despite these differences, the total interaction energy was again slightly larger for MIC(II). The stabilization due to the electrostatic (−104.1 kcal/mol) and orbital-interaction (−67.4 kcal/mol) effects is larger in MIC(II) than in MIC(III). At first glance, these results were counterintuitive in view of the smaller formal positive charge of Fe in MIC(II) (*i.e.*, +2) than in MIC(III) (*i.e.*, +3). To better understand this trend, we attempted to make a fairer comparison, performing EDA for ^1^MIC(II)’, which has the same geometry as ^1^MIC(II) except that the Fe–N(nitrosomethane) distance in ^1^MIC(II)’ was elongated to 2.01279 Å, so that the Fe–N distances in ^1^MIC(II)’ and ^2^MIC(III) were equal. This elongation did not change the total interaction energy significantly (Table 2). Consistent with the argument based on the formal charge of Fe, the electrostatic stabilization in ^1^MIC(II)’ (−67.9 kcal/mol) was smaller than that in ^2^MIC(III) (−75.8 kcal/mol) by about 8 kcal/mol. The orbital interaction energies were not very different in these complexes. Interestingly, the Pauli repulsion energy for ^1^MIC(II)’ was smaller (90.0 kcal/mol) than that for ^2^MIC(III) (103.1 kcal/mol) by about 13 kcal/mol. Our EDA data therefore suggest that the major reason the MIC(II) forms a stronger coordination bond is its intrinsically smaller Pauli repulsion. The smaller Pauli repulsion allows the two interacting fragments to come closer to each other. As a result of the bond shortening in going from ^1^MIC(II)’ to ^1^MIC(II), the Pauli repulsion increases significantly to 148.0 kcal/mol; however, the attractive electrostatic and orbital-interaction terms also increase, with these stabilizing effects slightly surpassing the Pauli destabilization. It should also be noted that the electrostatic and Pauli effects are not sufficient to fully explain the formation of a MIC, because the sum of these energy terms is positive: 22.2 kcal/mol for ^1^MIC(II)’ and 27.4 kcal/mol for ^2^MIC(III). Clearly, the other effect, orbital interaction, plays an important role in stabilizing the complex.

## 3. Experimental Section

We usually use the B3LYP functional for geometry optimization when studying P450 reactions [27,29,45–47]. However, in this study, we encountered difficulties in optimizing the geometries of a few transition states. Therefore, the M06 functional [48] was used instead for geometry optimization, in conjunction with the SDD effective core potential basis set for Fe and the 6-31G* basis set for the other atoms (B1) [49,50]. Single-point energy calculations were performed for all intermediates and transition states, using the B3LYP functional and the 6-311+G(d,p) basis set (B2), while taking into account the somewhat polar nature of the enzyme active site with the IEFPCM self-consistent-reaction-field (SCRF) method (ɛ = 5.6968) [51]. Because previous studies showed that the doublet spin state is mostly the ground state in the reactions of amines [25,26,35–37,52], the doublet was considered in this study.

Besides analyzing the reaction mechanisms of MI formation, we also investigated the interaction between the nitrosoalkane MI and the heme in the MIC. The M06 functional tended to overestimate the stability of high-spin states of pentacoordinate P450 intermediates (Table S7). Therefore, we mainly used the B3LYP(SCRF)/B2//M06/B1 data for the discussion on MICs. B3LYP-D3 dispersion energy correction with the Becke-Johnson (BJ) damping was also attempted [53–57]. EDA was performed at the B3LYP/TZP level using the M06/B1-optimized geometries [58–61].

Gaussian 09 was used for almost all calculations [62], but the Amsterdam Density Functional (ADF) program was used for the EDA [63,64]. Chimera was used to draw molecular structures [65]. Full Ref. 62 is given in the Supporting Information.

## 4. Conclusions

A DFT study was undertaken to elucidate the mechanism of the P450-catalyzed conversion of NMH into a nitrosomethane intermediate that eventually causes P450 inhibition. Based on the energy data, we conclude that the pathways involving *H*-abstraction from the O–H or the N–H bond are more plausible than the *N*-oxidation and C–H activation pathways. *H*-abstraction from the O–H bond was found to be slightly more favorable than *H*-abstraction from the N–H bond. However, the latter might also occur depending on the initial configuration of *N*-alkylhydroxylamine in the active site of a P450. The analysis of the coordination bond of MICs showed that the binding energy of MIC(II) is greater than that of MIC(III). Additional EDA showed that the Pauli repulsion is intrinsically smaller in MIC(II) than in MIC(III), which appears to be the main reason the MIC(II) forms a somewhat tighter complex.

## Supplementary Information



## Figures and Tables

**Table 1.** Interaction energies (kcal/mol) calculated for the MIC(II) and MIC(III) a.

	ΔE(M06)	ΔE(B3LYP)	ΔE(B3LYP-D3) b
MIC(II)	−3.7	−2.1	−13.6
MIC(III)	6.8	−0.4	−10.5

a Obtained from B2(SCRF) single-point calculations on the M06/B1-optimized geometries, with the M06/B1 zero-point energy effect included. Δ*E* was calculated as *E*(MIC) − *E*([Fe(Por)(SH)]) − *E*(nitrosomethane);

b With B3LYP-D3(BJ) corrections.

**Table 2.** Summary of B3LYP/TZP-EDA-derived energy terms for several MICs (in kcal/mol).

Energy term	^1^MIC(II)	^1^MIC(II) a	^2^MIC(III)
Electrostatic	−104.1	−67.9	−75.8
Repulsion	148.0	90.0	103.1
Orbital interaction	−67.4	−45.1	−45.8
Total interaction	−23.6	−22.9	−18.4

a The Fe–N distance of the geometry of ^1^MIC(II) was elongated to 2.01279 Å (which is the same as the distance in ^2^MIC(III)), while keeping all the other internal coordinates unchanged.
