# Supplementary material for: Importance of H-Abstraction in the Final Step of Nitrosoalkane Formation in the Mechanism-Based Inactivation of Cytochrome P450 by Amine-Containing Drugs
Source: Int J Mol Sci. 2013 Dec 18;14(12):24692–705. doi: 10.3390/ijms141224692 (PMC3876136; doi:10.3390/ijms141224692)
Supplement: Supplementary file 1 [file ijms-14-24692-s001.pdf]

Supplementary Information

Contents

- **Complete list of authors of Gaussian 09**
- **Table S1:** Raw energy data obtained at the M06/B1 level
- **Table S2:** Raw energy data obtained at the M06(SCRF)/B2//M06/B1 level
- **Table S3:** Raw energy data obtained at the B3LYP(SCRF)/B2//M06/B1 level
- **Table S4:** Mulliken group spin populations
- **Table S5:** Mulliken group charges
- **Table S6:** Homolytic dissociation energy
- **Table S7:** Energy data for the MIC formation
- **Scheme S1:** Possibility of outer-sphere electron transfer
- **XYZ coordinates of optimized geometries**

Complete list of authors of Gaussian 09

Gaussian 09, revision B.01; M. J. Frisch, G. W. Trucks, H. B. Schlegel, G. E. Scuseria, M. A. Robb, J. R. Cheeseman, G. Scalmani, V. Barone, B. Mennucci, G. A. Petersson, H. Nakatsuji, M. Caricato, X. Li, H. P. Hratchian, A. F. Izmaylov, J. Bloino, G. Zheng, J. L. Sonnenberg, M. Hada, M. Ehara, K. Toyota, R. Fukuda, J. Hasegawa, M. Ishida, T. Nakajima, Y. Honda, O. Kitao, H. Nakai, T. Vreven, J. A. Montgomery, Jr., J. E. Peralta, F. Ogliaro, M. Bearpark, J. J. Heyd, E. Brothers, K. N. Kudin, V. N. Staroverov, T. Keith, R. Kobayashi, J. Normand, K. Raghavachari, A. Rendell, J. C. Burant, S. S. Iyengar, J. Tomasi, M. Cossi, N. Rega, J. M. Millam, M. Klene, J. E. Knox, J. B. Cross, V. Bakken, C. Adamo, J. Jaramillo, R. Gomperts, R. E. Stratmann, O. Yazyev, A. J. Austin, R. Cammi, C. Pomelli, J. W. Ochterski, R. L. Martin, K. Morokuma, V. G. Zakrzewski, G. A. Voth, P. Salvador, J. J. Dannenberg, S. Dapprich, A. D. Daniels, O. Farkas, J. B. Foresman, J. V. Ortiz, J. Cioslowski, and D. J. Fox, Gaussian, Inc., Wallingford, CT, USA, 2010.

Table S1. Raw energy data obtained at the M06/B1 level.

	ΔE [kcal/mol]	Δ(E+ZPE) [kcal/mol]	E [au]	ZPE [au]
**RCa**	0.0	0.0	−1756.414741	0.357489
**TS1a**	0.9	−2.0	−1756.413279	0.352849
**INT1a**	−25.8	−26.5	−1756.455886	0.356353
**TS2a**	−24.4	−25.2	−1756.453580	0.356094
**INT2a**	−28.4	−29.2	−1756.459967	0.356123
**INT2a’**	−28.0	−29.4	−1756.459384	0.355321
**TS3a**	−20.6	−25.3	−1756.447616	0.350030
**PROa**	−43.8	−44.8	−1756.484531	0.355869
**RCb**	2.3	2.2	−1756.411027	0.357343
**TS1b**	5.7	2.9	−1756.405688	0.353073
**INT1b**	−17.3	−18.8	−1756.442248	0.355056
**INT1b’**	−24.6	−25.8	−1756.454008	0.355615
**TS2b**	−19.8	−24.2	−1756.446316	0.350478
**PROb**	−44.0	−45.3	−1756.484898	0.355515
**TS1c**	9.8	8.7	−1756.399062	0.355628
**INT1c**	−54.9	−53.4	−1756.502264	0.359927
**RCd**	0.8	0.9	−1756.413535	0.357759
**TS1d**	8.4	5.4	−1756.401394	0.352718
**PROd**	−48.5	−48.3	−1756.492052	0.357877

Table S2. Raw energy data obtained at the M06(SCRF)/B2//M06/B1 level.

	**ΔE [kcal/mol]**	**Δ(E+ZPE) [kcal/mol]**	**E [au]**	**ZPE [au]**
**RCa**	0.0	0.0	−2896.499043	0.357489
**TS1a**	0.2	−2.7	−2896.498710	0.352849
**INT1a**	−25.0	−25.7	−2896.538924	0.356353
**TS2a**	−24.2	−25.0	−2896.537532	0.356094
**INT2a**	−26.7	−27.5	−2896.541513	0.356123
**INT2a’**	−27.2	−28.6	−2896.542387	0.355321
**TS3a**	−25.4	−30.1	−2896.539588	0.350030
**PROa**	−47.1	−48.1	−2896.574149	0.355869
**RCb**	2.2	2.1	−2896.495519	0.357343
**TS1b**	7.3	4.5	−2896.487381	0.353073
**INT1b**	−17.0	−18.5	−2896.526127	0.355056
**INT1b’**	−22.1	−23.2	−2896.534198	0.355615
**TS2b**	−17.1	−21.5	−2896.526280	0.350478
**PROb**	−47.1	−48.4	−2896.574158	0.355515
**TS1c**	12.2	11.0	−2896.479656	0.355628
**INT1c**	−31.5	−29.9	−2896.549192	0.359927
**RCd**	−0.5	−0.3	−2896.499799	0.357759
**TS1d**	10.2	7.2	−2896.482823	0.352718
**PROd**	−57.1	−56.9	−2896.590067	0.357877

Table S3. Raw energy data obtained at the B3LYP(SCRF)/B2//M06/B1 level.

	ΔE [kcal/mol]	Δ(E+ZPE) [kcal/mol]	E [au]	ZPE [au]
**RCa**	0.0	0.0	−2897.499259	0.357489
**TS1a**	2.7	−0.3	−2897.495033	0.352849
**INT1a**	−18.8	−19.5	−2897.529230	0.356353
**TS2a**	−18.6	−19.5	−2897.528965	0.356094
**INT2a**	−20.7	−21.5	−2897.532185	0.356123
**INT2a'**	−20.9	−22.2	−2897.532493	0.355321
**TS3a**	−22.0	−26.7	−2897.534303	0.350030
**PROa**	−41.3	−42.3	−2897.565024	0.355869
**RCb**	2.1	2.0	−2897.495937	0.357343
**TS1b**	6.6	3.9	−2897.488664	0.353073
**INT1b**	−13.9	−15.5	−2897.521461	0.355056
**INT1b'**	−15.6	−16.7	−2897.524047	0.355615
**TS2b**	−15.6	−20.0	−2897.524142	0.350478
**PROb**	−40.2	−41.4	−2897.563270	0.355515
**TS1c**	14.5	13.3	−2897.476131	0.355628
**INT1c**	−25.5	−24.0	−2897.539970	0.359927
**RCd**	2.9	3.0	−2897.494691	0.357759
**TS1d**	14.2	11.2	−2897.476705	0.352718
**PROd**	−51.2	−51.0	−2897.580884	0.357877

Table S4. Mulliken group spin populations.

(a) M06/B1

	Fe	O	Por	SH	Substrate
**RCa**	1.59	0.60	−0.70	−0.50	0.01
**TS1a**	1.82	0.46	−0.63	−0.20	−0.45
**INT1a**	2.28	0.18	−0.24	−0.25	−0.98
**TS2a**	2.29	0.17	−0.23	−0.25	−0.98
**INT2a**	2.34	0.16	−0.25	−0.27	−0.98
**INT2a’**	2.39	0.15	−0.27	−0.31	−0.96
**TS3a**	1.85	0.03	−0.19	−0.20	−0.49
**PROa**	1.20	0.00	−0.13	−0.07	0.00
**RCb**	1.55	0.63	−0.65	−0.51	−0.01
**TS1b**	1.87	0.29	−0.41	−0.29	−0.47
**INT1b**	2.23	0.21	−0.23	−0.21	−1.00
**INT1b’**	2.44	0.14	−0.27	−0.32	−0.99
**TS2b**	2.32	0.06	−0.31	−0.36	−0.71
**PROb**	1.23	0.00	−0.14	−0.10	0.00
**TS1c**	2.07	−0.24	−0.25	−0.15	−0.43
**INT1c**	1.21	−0.01	−0.14	−0.07	0.01
**RCd**	1.63	0.56	−0.64	−0.49	−0.07
**TS1d**	2.12	−0.06	−0.27	−0.27	−0.52
**PROd**	1.22	0.00	−0.14	−0.09	0.00

Table S4. *Cont.*

(b) M06(SCRF)/B2//M06/B1

	Fe	O	Por	SH	Substrate
**RCa**	1.50	0.70	−0.80	−0.39	−0.01
**TS1a**	1.62	0.54	−0.55	−0.11	−0.51
**INT1a**	2.18	0.20	−0.24	−0.17	−0.97
**TS2a**	2.19	0.19	−0.23	−0.17	−0.98
**INT2a**	2.28	0.17	−0.29	−0.19	−0.97
**INT2a’**	2.38	0.14	−0.34	−0.22	−0.96
**TS3a**	1.14	0.01	−0.11	−0.01	−0.03
**PROa**	1.12	0.00	−0.12	0.00	0.00
**RCb**	1.48	0.73	−0.78	−0.42	−0.01
**TS1b**	1.64	0.44	−0.44	−0.19	−0.45
**INT1b**	2.15	0.20	−0.25	−0.13	−0.98
**INT1b’**	2.40	0.15	−0.30	−0.25	−1.01
**TS2b**	2.16	0.07	−0.25	−0.27	−0.71
**PROb**	1.12	0.00	−0.11	−0.01	0.00
**TS1c**	1.79	−0.01	−0.18	−0.07	−0.53
**INT1c**	1.09	0.00	-0.11	0.00	0.01
**RCd**	1.52	0.68	−0.76	−0.41	−0.02
**TS1d**	1.91	0.03	−0.23	−0.18	−0.54
**PROd**	1.12	0.00	−0.11	−0.01	0.00

Table S4. *Cont.*

(c) B3LYP(SCRF)/B2//M06/B1

	**Fe**	**O**	**Por**	**SH**	**Substrate**
**RCa**	1.50	0.77	−0.87	−0.40	−0.01
**TS1a**	1.62	0.59	−0.56	−0.08	−0.57
**INT1a**	2.07	0.23	−0.27	−0.05	−0.98
**TS2a**	2.12	0.21	−0.28	−0.05	−1.00
**INT2a**	2.17	0.18	−0.31	−0.06	−0.98
**INT2a’**	2.23	0.15	−0.32	−0.08	−0.97
**TS3a**	1.08	0.00	−0.10	0.05	−0.02
**PROa**	1.09	0.00	−0.14	0.05	0.00
**RCb**	1.45	0.80	−0.82	−0.42	−0.01
**TS1b**	1.64	0.50	−0.46	−0.15	−0.52
**INT1b**	2.01	0.24	−0.24	−0.01	−1.01
**INT1b’**	2.29	0.14	−0.32	−0.11	−1.01
**TS2b**	1.16	0.01	0.09	−0.01	−0.25
**PROb**	1.07	0.00	−0.13	0.06	0.00
**TS1c**	1.77	0.06	−0.26	−0.01	−0.56
**INT1c**	1.07	0.01	−0.14	0.05	0.00
**RCd**	1.52	0.75	−0.82	−0.42	−0.03
**TS1d**	1.91	0.11	−0.34	−0.11	−0.58
**PROd**	1.09	0.00	−0.13	0.04	0.00

Table S5. Mulliken group charges.

(a) M06/B1

	**Fe**	**O**	**Por**	**SH**	**Substrate**
**RCa**	0.49	−0.48	0.00	−0.04	0.03
**TS1a**	0.44	−0.63	−0.18	−0.09	0.46
**INT1a**	0.40	−0.69	−0.28	0.07	0.49
**TS2a**	0.37	−0.68	−0.27	0.07	0.52
**INT2a**	0.40	−0.71	−0.25	0.08	0.47
**INT2a’**	0.38	−0.72	−0.21	0.10	0.45
**TS3a**	0.31	−0.78	−0.34	0.08	0.73
**PROa**	0.21	−0.77	−0.51	0.08	1.00
**RCb**	0.49	−0.45	−0.04	−0.03	0.04
**TS1b**	0.42	−0.54	−0.22	−0.05	0.40
**INT1b**	0.36	−0.68	−0.27	0.05	0.54
**INT1b’**	0.36	−0.74	−0.20	0.11	0.47
**TS2b**	0.34	−0.81	−0.26	0.13	0.60
**PROb**	0.21	−0.77	−0.52	0.08	1.00
**TS1c**	0.37	−0.49	−0.33	−0.01	0.46
**INT1c**	−1.50	0.08	0.37	0.52	0.53
**RCd**	0.48	−0.49	−0.03	−0.03	0.08
**TS1d**	0.43	−0.59	−0.26	0.00	0.41
**PROd**	0.22	−0.79	−0.51	0.08	0.99

Table S5. *Cont.*

(b) M06(SCRF)/B2//M06/B1

	**Fe**	**O**	**Por**	**SH**	**substrate**
**RCa**	−1.04	−0.36	1.94	−0.63	0.09
**TS1a**	−0.89	−0.47	1.64	−0.80	0.52
**INT1a**	−1.75	−0.40	2.50	−0.65	0.30
**TS2a**	−1.25	−0.35	2.05	−0.69	0.24
**INT2a**	−0.73	−0.39	1.51	−0.64	0.25
**INT2a’**	−0.75	−0.50	1.58	−0.65	0.31
**TS3a**	0.14	−0.65	0.55	−0.90	0.86
**PROa**	−0.58	−0.34	1.04	−0.76	0.64
**RCb**	−0.83	−0.36	1.70	−0.64	0.13
**TS1b**	−1.09	−0.36	1.70	−0.75	0.50
**INT1b**	−1.84	−0.37	2.46	−0.58	0.33
**INT1b’**	−1.35	−0.54	2.17	−0.60	0.32
**TS2b**	−0.47	−0.56	1.35	−0.70	0.39
**PROb**	−1.05	−0.26	1.36	−0.71	0.65
**TS1c**	−1.78	−0.39	2.37	−0.68	0.48
**INT1c**	−0.57	−0.01	1.01	−0.63	0.20
**RCd**	−1.04	−0.35	1.90	−0.60	0.08
**TS1d**	−0.93	−0.42	1.62	−0.73	0.46
**PROd**	−1.05	−0.42	1.51	−0.75	0.71

Table S5. *Cont.*

(c) B3LYP(SCRF)/B2//M06/B1

	Fe	O	Por	SH	substrate
**RCa**	−1.43	−0.28	2.31	−0.70	0.09
**TS1a**	−1.38	−0.40	2.07	−0.86	0.57
**INT1a**	−2.22	−0.32	2.93	−0.69	0.30
**TS2a**	−1.73	−0.30	2.47	−0.72	0.28
**INT2a**	−1.30	−0.30	2.03	−0.67	0.24
**INT2a’**	−1.13	−0.39	1.97	−0.69	0.23
**TS3a**	−0.18	−0.60	0.99	−0.95	0.74
**PROa**	−0.79	−0.30	1.33	−0.81	0.57
**RCb**	−1.31	−0.26	2.13	−0.70	0.14
**TS1b**	−1.64	−0.25	2.13	−0.81	0.57
**INT1b**	−2.33	−0.39	2.92	−0.63	0.43
**INT1b’**	−1.70	−0.43	2.51	−0.63	0.25
**TS2b**	−0.62	−0.58	1.44	−0.94	0.70
**PROb**	−1.22	−0.22	1.63	−0.75	0.55
**TS1c**	−2.18	−0.22	2.70	−0.75	0.46
**INT1c**	−0.80	0.07	1.25	−0.67	0.15
**RCd**	−1.51	−0.24	2.31	−0.66	0.10
**TS1d**	−1.53	−0.30	2.13	−0.79	0.49
**PROd**	−1.35	−0.38	1.88	−0.79	0.63

Table S6. Homolytic dissociation energy.

(a) M06/B1

	**E [au]**	**ZPE [au]**
Substrate	−170.909330	0.069055
Hydrogen atom	−0.497919	0.000000
C-radical	−170.246410	0.054535
N-radical	−170.278153	0.055325
O-radical	−170.289185	0.055698

(b) M06(SCRF)/B2//M06/B1

	**E [au]**	**ZPE [au]**
Substrate	−170.977699	0.069055
Hydrogen atom	−0.499894	0.000000
C-radical	−170.316580	0.054535
N-radical	−170.341834	0.055325
O-radical	−170.354757	0.055698

(c) B3LYP(SCRF)/B2//M06/B1

	**E [au]**	**ZPE [au]**
Substrate	−171.091355	0.069055
Hydrogen atom	−0.502174	0.000000
C-radical	−170.430894	0.054535
N-radical	−170.455610	0.055325
O-radical	−170.470556	0.055698

(d) Bond dissociation energy

	**M06/B1 [kcal/mol]**	**M06(SCRF)/B2 //M06/B1 [kcal/mol]**	**B3LYP(SCRF)/B2 //M06/B1 [kcal/mol]**
O–H bond	68.3	68.8	66.1
N–H bond	75.0	76.7	75.2
C–H bond	94.4	92.1	90.2

Table S7. Energy data for the MIC formation.

(a) M06/B1

	E [au]	ZPE [au]
^{1}[Fe(II)(Por)SH]	−1510.431413	0.282631
^{5}[Fe(II)(Por)SH]	−1510.493827	0.278476
^{2}[Fe(III)(Por)SH]	−1510.380705	0.284859
^{6}[Fe(III)(Por)SH]	−1510.429771	0.282962
nitrosomethane	−169.686295	0.043344
^{1}MIC(II), N-bound	−1680.170716	0.329955
^{2}MIC(III), N-bound	−1680.099546	0.330849
^{1}MIC(II), O-bound	−1680.144894	0.328153
^{2}MIC(III), O-bound	−1680.085446	0.329466

(b) M06(SCRF)/B2//M06/B1

	E [au]	ZPE [au]
^{1}[Fe(II)(Por)SH]	−2650.499887	0.282631
^{5}[Fe(II)(Por)SH]	−2650.535686	0.278476
^{2}[Fe(III)(Por)SH]	−2650.378398	0.284859
^{6}[Fe(III)(Por)SH]	−2650.412653	0.282962
nitrosomethane	−169.743770	0.043344
^{1}MIC(II), N-bound	−2820.293417	0.329955
^{2}MIC(III), N-bound	−2820.150151	0.330849
^{1}MIC(II), O-bound	−2820.266030	0.328153
^{2}MIC(III), O-bound	−2820.136354	0.329466

(c) B3LYP(SCRF)/B2//M06/B1

	E [au]	ZPE [au]
^{1}[Fe(II)(Por)SH]	−2651.357412	0.282631
^{5}[Fe(II)(Por)SH]	−2651.378144	0.278476
^{2}[Fe(III)(Por)SH]	−2651.236956	0.284859
^{6}[Fe(III)(Por)SH]	−2651.243553	0.282962
nitrosomethane	−169.854083	0.043344
^{1}MIC(II), N-bound	−2821.243632	0.329955
^{2}MIC(III), N-bound	−2821.102857	0.330849
^{1}MIC(II), O-bound	−2821.223045	0.328153
^{2}MIC(III), O-bound	−2821.094733	0.329466

Scheme S1. Possibility of outer-sphere electron transfer.

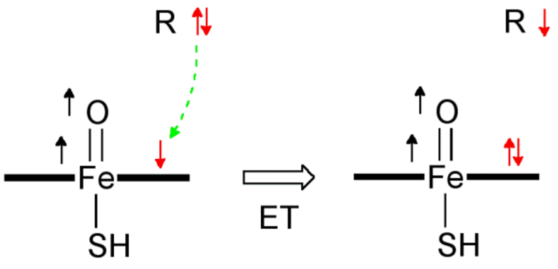

RCa **RCa'**

The Cpd I moiety within the **RC** state has a triradicaloid character, while the substrate has a closed-shell character (see **RCa** above). We attempted to obtain a state that has a closed-shell porphine ligand and a substrate radical. However, attempts to obtain such an electron-transferred state failed.

XYZ coordinates of optimized geometry (in Å)

=== RCa ===

H	11.205085	33.165112	1.559338
S	10.007782	32.984316	2.160978
Fe	10.029972	30.484955	2.047414
N	10.956236	30.585465	0.278413
N	11.793405	30.766377	2.966759
N	9.101867	30.662416	3.818504
N	8.256519	30.527133	1.123916
C	10.375541	30.538874	−0.955790
C	11.373832	30.510632	−1.986079
C	12.576819	30.530076	−1.357863
C	12.300475	30.579570	0.049752
C	13.033022	30.761494	2.387337
C	14.059009	30.860968	3.386518
C	13.428812	30.898934	4.587350
C	12.021159	30.827868	4.315461
C	9.682572	30.720239	5.052023
C	8.684091	30.704268	6.083908
C	7.482278	30.633709	5.459652
C	7.755019	30.602908	4.049993
C	7.025020	30.497859	1.703644
C	5.993341	30.453137	0.707050
C	6.620929	30.458482	−0.496602
C	8.028843	30.502155	−0.220596
C	9.011978	30.514523	−1.195607
H	8.687090	30.489119	−2.235243
C	13.276082	30.659812	1.031159
H	14.315967	30.649650	0.707126
C	11.044439	30.795806	5.290934
H	11.370653	30.828064	6.329387
C	6.783849	30.527597	3.069928
H	5.744627	30.494069	3.395274
H	13.572309	30.519321	−1.788958
H	11.159164	30.478037	−3.049041
H	15.122277	30.893829	3.171757
H	13.857620	30.968642	5.581426
H	8.899953	30.745206	7.146242
H	6.488495	30.603394	5.893816
H	4.930439	30.426509	0.922846
H	6.189264	30.434925	−1.491658
O	11.983795	27.700700	3.829264
N	13.121176	27.605708	2.985745
C	13.483924	26.201969	2.909068
H	12.722678	25.668643	2.328967
H	14.444580	26.117403	2.383382
H	13.566871	25.720096	3.898069
H	13.847212	28.094989	3.512188
H	11.291491	28.059117	3.229620
O	10.100180	28.853581	2.138258

=== TS1a ===

H	11.164957	32.957522	1.382894
S	9.943490	32.802062	1.942021
Fe	10.137561	30.435844	2.312574
N	11.164869	30.300356	0.588890
N	11.854212	30.803175	3.282027
N	9.102146	30.765747	3.998354
N	8.410844	30.236544	1.303736
C	10.642123	30.087226	−0.658413
C	11.695564	29.953439	−1.623692
C	12.865258	30.074799	−0.941865
C	12.514834	30.289569	0.433444
C	13.113521	30.755336	2.780086
C	14.081681	31.015355	3.808293
C	13.382762	31.228551	4.951797
C	11.993927	31.090383	4.616183
C	9.611683	31.049260	5.225343
C	8.560455	31.142101	6.200365
C	7.399296	30.902309	5.539797
C	7.751662	30.669992	4.166555
C	7.153474	30.217702	1.825459
C	6.181243	29.999689	0.790511
C	6.873383	29.893470	−0.372023
C	8.262267	30.041517	−0.034743
C	9.296562	29.977734	−0.956114
H	9.027705	29.809314	−1.998460
C	13.436496	30.453310	1.462846
H	14.493048	30.431870	1.197589
C	10.960346	31.210753	5.519642
H	11.221654	31.436696	6.552834
C	6.837884	30.409404	3.161359
H	5.785189	30.368415	3.438891
H	13.881769	30.035107	−1.320398
H	11.538762	29.789564	−2.684879
H	15.155495	31.036257	3.652022
H	13.753567	31.461547	5.944433
H	8.715462	31.361888	7.251531
H	6.385567	30.882923	5.926036
H	5.110109	29.945070	0.954592
H	6.497719	29.730423	−1.376789
O	12.383157	27.905669	3.483101
N	13.127783	27.843319	2.356433

C	12.522894	27.104083	1.267284
H	11.590773	27.614199	0.987230
H	13.213786	27.108574	0.417294
H	12.284170	26.069744	1.560566
H	14.061940	27.541022	2.624014
H	11.427942	28.290673	3.173457
O	10.236745	28.778157	2.602922

=== INT1a ===

H	10.354450	32.658150	0.681991
S	10.007123	32.719425	1.987880
Fe	10.255970	30.422435	2.421690
N	11.330507	30.251642	0.736185
N	11.934688	30.805425	3.436379
N	9.176769	30.813450	4.056438
N	8.581811	30.072083	1.388726
C	10.872389	29.806968	−0.475433
C	11.954874	29.740040	−1.413187
C	13.069653	30.166699	−0.760215
C	12.670814	30.469742	0.584591
C	13.195694	30.945879	2.928611
C	14.127960	31.211597	3.987598
C	13.415569	31.227892	5.141586
C	12.043527	31.001526	4.784568
C	9.653892	31.014725	5.318713
C	8.561751	31.181442	6.236339
C	7.420079	31.079692	5.509025
C	7.814211	30.828377	4.151453
C	7.300731	30.184568	1.845089
C	6.375088	29.822582	0.808951
C	7.116355	29.483343	−0.276927
C	8.493100	29.659905	0.091411
C	9.557322	29.503098	−0.779253
H	9.337377	29.167679	−1.791641
C	13.548362	30.828492	1.597021
H	14.593934	30.983177	1.332752
C	10.989708	31.072698	5.676581
H	11.225329	31.239394	6.726298
C	6.929533	30.562108	3.123069
H	5.865178	30.606905	3.347951
H	14.082403	30.267079	−1.137953
H	11.852817	29.418506	−2.444679
H	15.193401	31.356925	3.842705
H	13.763706	31.398640	6.154832
H	8.678643	31.356661	7.300732
H	6.390386	31.144370	5.844825
H	5.296053	29.828417	0.922368
H	6.780630	29.155508	−1.255360
O	13.013361	27.779024	3.262641
N	13.208977	27.586652	2.022040
C	12.341590	26.696656	1.279343
H	11.297899	27.031176	1.381399
H	12.640929	26.709070	0.225724
H	12.435730	25.680973	1.682188
H	13.714271	28.322724	1.517892
H	11.182591	28.422559	3.246367
O	10.335127	28.653003	2.806884

=== TS2a ===

H	10.229656	32.814959	0.559834
S	10.063652	32.937643	1.896529
Fe	10.297826	30.647180	2.365403
N	11.336786	30.442372	0.661144
N	11.999783	31.026796	3.349980
N	9.255484	31.059814	4.024712
N	8.602906	30.290723	1.371803
C	10.845745	30.036039	−0.550325
C	11.909643	29.958057	−1.509342
C	13.048114	30.328320	−0.865313
C	12.682718	30.613769	0.492023
C	13.257659	31.102178	2.819819
C	14.213515	31.376039	3.853793
C	13.519219	31.469345	5.015638
C	12.136066	31.271627	4.688303
C	9.758313	31.310171	5.268640
C	8.685620	31.491037	6.205854
C	7.529089	31.344973	5.510230
C	7.895654	31.059273	4.151962
C	7.332877	30.389644	1.864206
C	6.382377	30.034049	0.849639
C	7.095237	29.720520	−0.262816
C	8.480079	29.899674	0.069456
C	9.519935	29.758484	−0.831909
H	9.272742	29.446181	−1.845379
C	13.586337	30.937075	1.488591
H	14.634635	31.032409	1.210154
C	11.100174	31.387926	5.596247
H	11.357033	31.591697	6.634390
C	6.989931	30.764992	3.150041
H	5.930909	30.799767	3.400486
H	14.059043	30.396281	−1.254147
H	11.779082	29.660245	−2.544724
H	15.281085	31.475819	3.688165
H	13.888096	31.669843	6.016068

H	8.825315	31.703381	7.260778
H	6.506491	31.404950	5.867991
H	5.306901	30.030212	0.993488
H	6.734795	29.406096	−1.236883
O	12.990731	27.898078	3.515806
N	12.737840	26.942943	2.716239
C	12.782409	27.180006	1.289100
H	13.762979	27.599199	1.037677
H	12.635107	26.233004	0.759454
H	11.993965	27.897800	1.010622
H	12.114751	26.207989	3.063781
H	11.232577	28.749247	3.280089
O	10.403199	28.881465	2.777131

=== INT2a ===

H	10.199651	32.821527	0.770744
S	9.823741	32.889950	2.067912
Fe	10.332440	30.656033	2.589034
N	11.503524	30.574610	0.966953
N	11.905796	31.266440	3.677969
N	9.130889	30.935415	4.162998
N	8.764656	30.068290	1.491271
C	11.154649	30.093146	−0.266879
C	12.293857	30.097819	−1.137284
C	13.338170	30.580410	−0.414681
C	12.839286	30.863524	0.898406
C	13.178016	31.503403	3.233832
C	14.000158	31.965377	4.314816
C	13.207812	32.016853	5.415985
C	11.898868	31.597086	5.004964
C	9.508203	31.282111	5.429480
C	8.364498	31.306970	6.295007
C	7.290760	30.969552	5.535654
C	7.780404	30.727512	4.209132
C	7.462469	29.992230	1.896393
C	6.637709	29.524816	0.819136
C	7.458144	29.321671	−0.243571
C	8.781826	29.677636	0.182368
C	9.893556	29.664595	−0.641083
H	9.760511	29.320710	−1.665967
C	13.624598	31.321816	1.939774
H	14.673679	31.523689	1.731550
C	10.794803	31.583772	5.836024
H	10.943979	31.859623	6.878605
C	6.989864	30.305268	3.156895
H	5.922993	30.189725	3.340595
H	14.368199	30.729884	−0.721067
H	12.274882	29.766289	−2.170691
H	15.052295	32.212488	4.219827
H	13.463100	32.320292	6.425951
H	8.401745	31.555275	7.350548
H	6.250766	30.875090	5.830110
H	5.565066	29.377548	0.890031
H	7.208040	28.976298	−1.241435
O	13.215908	27.928557	2.079219
N	12.312851	27.592670	1.247071
C	12.637436	26.735749	0.137071
H	12.808097	25.698335	0.462989
H	11.813714	26.757070	−0.585990
H	13.553307	27.108272	−0.337791
H	11.343871	27.810402	1.520246
H	11.600440	28.827005	3.265072
O	10.654848	28.906490	3.027226

=== INT2a’ ===

H	10.927896	33.088022	1.265391
S	9.839448	32.852342	2.032643
Fe	10.420683	30.645205	2.604924
N	11.628544	30.591047	1.017984
N	11.924551	31.347574	3.723989
N	9.161874	30.910142	4.138297
N	8.923178	29.943377	1.486284
C	11.292337	30.238309	−0.261910
C	12.457765	30.247356	−1.098088
C	13.509869	30.560107	−0.300652
C	12.983827	30.781107	1.015348
C	13.233246	31.482591	3.345428
C	13.996227	32.058270	4.413778
C	13.127291	32.308520	5.428096
C	11.836331	31.867077	4.989904
C	9.448914	31.466044	5.354220
C	8.291020	31.424508	6.198185
C	7.308278	30.811890	5.486891
C	7.860515	30.497601	4.201978
C	7.653188	29.662108	1.909262
C	6.867149	29.173467	0.812947
C	7.664905	29.201911	−0.285951
C	8.944575	29.687942	0.144014
C	10.037639	29.845974	−0.688554
H	9.915020	29.597089	−1.741565
C	13.745203	31.181200	2.096429
H	14.815345	31.305831	1.942790
C	10.687397	31.936247	5.754011
H	10.769295	32.361108	6.753121

C	7.155827	29.888756	3.178162
H	6.119472	29.616555	3.371317
H	14.561371	30.633716	−0.555774
H	12.452767	30.002575	−2.155311
H	15.061864	32.256250	4.365653
H	13.323638	32.752401	6.398616
H	8.259601	31.806537	7.213223
H	6.289976	30.586549	5.786246
H	5.827649	28.872555	0.891735
H	7.427281	28.924412	−1.307721
O	13.413469	27.862885	1.188079
N	12.185638	27.572085	1.034451
C	11.795233	26.685306	−0.031035
H	12.195759	25.673245	0.130823
H	10.701186	26.638491	−0.080754
H	12.190498	27.062227	−0.985168
H	11.514480	27.933360	1.734630
H	11.444540	28.936547	3.838320
O	10.792318	28.900877	3.119734

=== TS3a ===

H	11.068173	33.316015	1.958760
S	9.833168	32.769672	1.870645
Fe	10.442495	30.668731	2.534064
N	11.680949	30.538681	0.974068
N	11.919742	31.387962	3.643592
N	9.211258	30.795606	4.105495
N	8.967228	29.937400	1.427523
C	11.346132	30.205821	−0.315873
C	12.512735	30.204478	−1.144861
C	13.564462	30.512021	−0.339343
C	13.027751	30.746562	0.971603
C	13.238521	31.521593	3.282610
C	13.990715	32.114575	4.352819
C	13.113116	32.363174	5.356850
C	11.828033	31.899972	4.913363
C	9.471956	31.374335	5.313459
C	8.320450	31.290229	6.165181
C	7.360189	30.638484	5.458580
C	7.922943	30.348487	4.171022
C	7.713008	29.594988	1.850854
C	6.928203	29.112251	0.747404
C	7.710960	29.195752	−0.357576
C	8.982332	29.706348	0.076114
C	10.078099	29.858652	−0.751657
H	9.951848	29.628396	−1.808937
C	13.773004	31.184062	2.055299
H	14.843449	31.321484	1.912038
C	10.688092	31.916770	5.693524
H	10.763203	32.347890	6.690771
C	7.225968	29.751462	3.134573
H	6.199935	29.441680	3.327824
H	14.615499	30.597049	−0.594476
H	12.513298	29.971125	−2.204819
H	15.055696	32.318326	4.311866
H	13.298582	32.810133	6.328317
H	8.276142	31.677699	7.177853
H	6.350022	30.379733	5.758840
H	5.901165	28.771502	0.829929
H	7.472654	28.934928	−1.383697
O	13.359890	27.853849	1.119015
N	12.138256	27.703603	1.203957
C	11.470335	26.900254	0.192390
H	11.572677	25.836341	0.451277
H	10.407698	27.166294	0.174372
H	11.944078	27.083416	−0.779172
H	11.565209	28.206189	2.087535
H	11.470051	28.934239	3.853022
O	10.894708	28.841079	3.078425

=== PROa ===

H	10.253084	32.801114	0.682095
S	9.927708	32.842830	1.997351
Fe	10.221711	30.694030	2.452160
N	11.369393	30.400134	0.834089
N	11.869477	31.017810	3.550669
N	9.104584	30.895985	4.094767
N	8.617749	30.144412	1.402483
C	10.951918	30.059404	−0.425978
C	12.050312	30.105102	−1.350295
C	13.141778	30.497065	−0.638631
C	12.708024	30.670634	0.719645
C	13.150543	31.161174	3.084416
C	14.036677	31.520566	4.154609
C	13.276608	31.618217	5.275585
C	11.927316	31.327075	4.884144
C	9.522084	31.210639	5.356984
C	8.398990	31.308156	6.252318
C	7.292999	31.048272	5.513507
C	7.745198	30.786553	4.172099
C	7.325717	30.130337	1.843087
C	6.437157	29.748899	0.780204
C	7.208601	29.539685	−0.316730
C	8.564331	29.800879	0.080153

C	9.648620	29.747528	−0.778206
H	9.456346	29.480256	−1.816855
C	13.546958	31.013990	1.767240
H	14.597749	31.186934	1.536399
C	10.839042	31.400460	5.737900
H	11.032849	31.653951	6.779495
C	6.909225	30.435241	3.128237
H	5.840902	30.382757	3.334976
H	14.157388	30.661731	−0.986015
H	11.972578	29.883043	−2.410080
H	15.103698	31.682426	4.039928
H	13.580264	31.879115	6.284292
H	8.473782	31.545175	7.308667
H	6.254309	31.022235	5.826889
H	5.360413	29.659786	0.882331
H	6.906780	29.243574	−1.316286
O	13.359790	27.798514	2.773392
N	12.742261	27.575419	1.762729
C	13.574259	27.204357	0.614900
H	14.632040	27.157146	0.896899
H	13.198997	26.245780	0.235788
H	13.387361	27.962597	−0.160374
H	10.951468	28.222711	2.290291
H	10.966896	28.675901	3.760956
O	10.388143	28.629691	2.982197

=== RCb ===

H	11.038186	33.224348	1.427119
S	9.982952	32.906238	2.210306
Fe	10.172652	30.431029	1.919802
N	10.918273	30.716552	0.084377
N	11.986183	30.812928	2.700221
N	9.397934	30.424007	3.770381
N	8.327922	30.358642	1.152242
C	10.234501	30.692596	−1.098131
C	11.135574	30.818355	−2.207691
C	12.383918	30.911050	−1.682288
C	12.232165	30.848887	−0.256820
C	13.165331	30.943826	2.016203
C	14.267381	31.068140	2.929706
C	13.746187	30.991111	4.178805
C	12.328514	30.818407	4.024897
C	10.080101	30.475105	4.951582
C	9.183643	30.307901	6.061583
C	7.943656	30.145486	5.536613
C	8.091181	30.213925	4.110092
C	7.163438	30.169288	1.833869
C	6.051878	30.093270	0.930568
C	6.563252	30.241663	−0.321401
C	7.980355	30.404087	−0.163803
C	8.863384	30.559031	−1.220822
H	8.446633	30.558913	−2.227036
C	13.286054	30.949996	0.640402
H	14.287744	31.054852	0.224883
C	11.446935	30.656030	5.076629
H	11.860473	30.670165	6.084294
C	7.046780	30.090490	3.212946
H	6.050609	29.928741	3.623117
H	13.334291	31.019891	−2.194374
H	10.831082	30.828894	−3.248863
H	15.302302	31.194541	2.629608
H	14.256728	31.040548	5.134875
H	9.487141	30.317682	7.103072
H	6.999642	29.992039	6.048901
H	5.019638	29.952616	1.233741
H	6.043860	30.255672	−1.274233
O	7.698051	27.203901	0.500043
N	8.925415	27.386479	−0.208782
C	9.417517	26.060832	−0.520777
H	8.779691	25.599732	−1.285160
H	10.432267	26.162684	−0.925484
H	9.446489	25.393421	0.356653
H	9.543624	27.813085	0.492885
H	7.097005	27.820166	0.054013
O	10.429070	28.819028	1.911629

=== TS1b ===

H	10.757146	32.832306	3.099902
S	9.964455	32.848420	2.004495
Fe	9.759749	30.460310	1.806720
N	10.397759	30.606768	−0.094912
N	11.658505	30.274225	2.427770
N	9.148333	30.487058	3.713973
N	7.888110	30.845878	1.197064
C	9.621025	30.734956	−1.209421
C	10.434201	30.701369	−2.389596
C	11.724258	30.560122	−1.970562
C	11.686002	30.510163	−0.535921
C	12.776247	30.219806	1.647549
C	13.942447	29.998045	2.456440
C	13.512920	29.908407	3.740402
C	12.086471	30.079479	3.709520
C	9.906980	30.254792	4.823129
C	9.085628	30.271809	6.003704

C	7.818092	30.518704	5.591595
C	7.867112	30.645575	4.159600
C	6.781920	30.964040	1.982779
C	5.609419	31.157284	1.173626
C	6.021736	31.139611	−0.117861
C	7.446061	30.943913	−0.091070
C	8.242093	30.881807	−1.218344
H	7.753160	30.973770	−2.187300
C	12.800252	30.322344	0.267258
H	13.768468	30.246436	−0.225738
C	11.276835	30.059600	4.832090
H	11.755570	29.892302	5.796076
C	6.762128	30.880511	3.364858
H	5.798775	30.990072	3.860997
H	12.630823	30.511326	−2.565302
H	10.053883	30.789066	−3.402174
H	14.952437	29.920161	2.067443
H	14.091223	29.743151	4.643559
H	9.458212	30.116256	7.010893
H	6.913291	30.608039	6.183638
H	4.606806	31.282844	1.568943
H	5.434888	31.250233	−1.023736
O	10.638962	27.474174	−1.132625
N	11.088250	27.492059	0.167391
C	10.990503	26.155460	0.691858
H	11.695153	25.499632	0.165940
H	11.241217	26.201563	1.755887
H	9.973529	25.749177	0.575536
H	10.508564	28.184639	0.773435
H	10.857826	28.360856	−1.483588
O	9.520182	28.797750	1.714465

=== INT1b ===

H	11.074795	33.070549	2.532359
S	9.842930	32.904564	1.999568
Fe	9.893288	30.562061	1.919683
N	10.553824	30.662088	0.019092
N	11.786306	30.588192	2.575660
N	9.240119	30.482515	3.807701
N	8.007224	30.750168	1.264933
C	9.790427	30.775028	−1.107289
C	10.630521	30.802768	−2.269690
C	11.917694	30.718530	−1.825387
C	11.854994	30.644957	−0.394118
C	12.920277	30.563748	1.810012
C	14.081870	30.489762	2.649827
C	13.636617	30.453804	3.931338
C	12.202709	30.500813	3.873173
C	10.000721	30.428129	4.938725
C	9.157298	30.382296	6.101477
C	7.877096	30.425239	5.656365
C	7.940177	30.499916	4.222153
C	6.876382	30.719807	2.027287
C	5.712711	30.808319	1.190766
C	6.155835	30.880711	−0.089635
C	7.590338	30.835268	−0.034139
C	8.408562	30.850293	−1.146499
H	7.933891	30.928976	−2.123227
C	12.966053	30.587891	0.429790
H	13.946190	30.567878	−0.044007
C	11.381433	30.437581	4.984792
H	11.856859	30.379838	5.962550
C	6.831164	30.604605	3.405583
H	5.851300	30.601928	3.880185
H	12.837989	30.729736	−2.400815
H	10.266579	30.898977	−3.287921
H	15.102038	30.462352	2.281130
H	14.209293	30.389405	4.850706
H	9.524216	30.329388	7.121237
H	6.955271	30.416789	6.228612
H	4.692590	30.807717	1.560399
H	5.581322	30.951403	−1.007398
O	10.884782	27.549075	−1.949860
N	10.405822	27.422421	−0.687084
C	10.191637	26.020792	−0.464849
H	11.132919	25.457389	−0.567322
H	9.798788	25.900405	0.549654
H	9.473435	25.611030	−1.192991
H	10.084446	28.480655	0.929685
H	11.014748	28.512734	−2.061057
O	9.840221	28.764322	1.839462

=== INT1b’ ===

H	0.250807	2.580218	−1.013257
S	−0.103083	2.488058	0.288464
Fe	−0.178987	0.131371	0.360098
N	0.338518	0.087093	−1.566112
N	1.750096	0.067488	0.877874
N	−0.695014	0.415151	2.273806
N	−2.120726	0.188185	−0.162755
C	−0.507067	−0.004921	−2.640238
C	0.247345	−0.110256	−3.853740
C	1.559854	−0.086227	−3.505552
C	1.607904	0.019672	−2.077615

C	2.829713	0.028049	0.039653
C	4.045675	0.013742	0.800218
C	3.689329	0.060767	2.109254
C	2.256276	0.114050	2.147357
C	0.144332	0.396129	3.353045
C	−0.606299	0.558007	4.563508
C	−1.911479	0.670524	4.204826
C	−1.958812	0.566932	2.775740
C	−3.189650	0.408414	0.665458
C	−4.402930	0.446736	−0.096659
C	−4.056404	0.270218	−1.398194
C	−2.630228	0.133071	−1.432441
C	−1.887649	0.015010	−2.591579
H	−2.427094	−0.044635	−3.535190
C	2.776668	0.011526	−1.340849
H	3.718189	−0.037035	−1.884837
C	1.518243	0.246484	3.307389
H	2.059411	0.260534	4.251952
C	−3.126480	0.578910	2.035300
H	−4.063400	0.726120	2.569943
H	2.435980	−0.151599	−4.142249
H	−0.194986	−0.198376	−4.840698
H	5.038489	−0.027315	0.364642
H	4.324865	0.072171	2.988565
H	−0.168294	0.578816	5.555937
H	−2.783276	0.799637	4.837594
H	−5.388487	0.597587	0.331566
H	−4.693888	0.247949	−2.275848
O	1.050664	−3.032978	−1.399916
N	−0.034940	−3.144507	−2.202572
C	0.435430	−3.755201	−3.416483
H	0.863548	−4.753063	−3.222614
H	−0.408747	−3.851790	−4.107891
H	1.224029	−3.142287	−3.887179
H	−1.154151	−2.001802	0.275653
H	0.685252	−2.574137	−0.592126
O	−0.258735	−1.724487	0.533516

=== TS2b ===

H	0.839464	2.555393	−0.723154
S	−0.127444	2.465938	0.218225
Fe	−0.118350	0.121629	0.333887
N	0.432639	0.032112	−1.575615
N	1.800731	0.149226	0.870422
N	−0.680866	0.340092	2.240008
N	−2.046141	0.094810	−0.207222
C	−0.396306	−0.062598	−2.662738
C	0.371830	−0.157469	−3.868319
C	1.679769	−0.135296	−3.504406
C	1.709187	−0.019241	−2.075087
C	2.898316	0.097276	0.054137
C	4.100814	0.141671	0.831625
C	3.720629	0.231361	2.132715
C	2.288033	0.244412	2.147228
C	0.148601	0.413476	3.327556
C	−0.620186	0.556518	4.527396
C	−1.927812	0.572401	4.158228
C	−1.954030	0.434944	2.732162
C	−3.141050	0.248982	0.603313
C	−4.344065	0.259923	−0.179523
C	−3.969088	0.137263	−1.476995
C	−2.537078	0.052036	−1.489410
C	−1.780783	−0.035715	−2.635892
H	−2.304854	−0.088147	−3.588880
C	2.867835	0.020515	−1.326385
H	3.818191	−0.025462	−1.855245
C	1.528321	0.358097	3.295244
H	2.057278	0.425758	4.244728
C	−3.112478	0.393925	1.975826
H	−4.063436	0.494978	2.496179
H	2.564122	−0.190324	−4.130652
H	−0.059421	−0.240365	−4.860939
H	5.101327	0.106004	0.413516
H	4.341310	0.286156	3.021103
H	−0.192262	0.633599	5.521628
H	−2.811701	0.662798	4.780839
H	−5.341212	0.360735	0.236440
H	−4.589739	0.115019	−2.366724
O	0.749805	−2.900844	−1.407853
N	−0.337520	−2.913259	−2.128741
C	−0.039191	−3.560096	−3.385538
H	0.217203	−4.619019	−3.220027
H	−0.919636	−3.494650	−4.034341
H	0.825354	−3.077975	−3.871641
H	−1.036948	−2.111157	0.602448
H	0.400656	−2.411045	−0.439450
O	−0.110006	−1.826383	0.574689

=== PROb ===

H	9.192370	33.175033	1.410997
S	10.373891	32.756059	1.925346
Fe	9.922162	30.586071	1.992588
N	10.549783	30.299157	0.105435
N	11.777462	30.228389	2.652798

N	9.265040	30.684065	3.871543
N	8.050075	30.818291	1.325362
C	9.800708	30.402419	−1.035672
C	10.632654	30.256389	−2.195258
C	11.904291	30.078557	−1.742896
C	11.840609	30.119779	−0.310682
C	12.904702	30.039165	1.899971
C	14.057737	29.882929	2.746007
C	13.617101	29.985095	4.024021
C	12.196963	30.205968	3.952858
C	10.018287	30.608948	5.008486
C	9.199815	30.826774	6.169404
C	7.939974	31.051912	5.719362
C	7.994620	30.966586	4.285692
C	6.934937	31.076281	2.075615
C	5.785538	31.252423	1.230012
C	6.214859	31.099785	−0.047690
C	7.626808	30.835551	0.021666
C	8.434450	30.632438	−1.082933
H	7.966638	30.686249	−2.065604
C	12.943518	29.989532	0.519000
H	13.913805	29.840487	0.046553
C	11.381483	30.377517	5.058016
H	11.850540	30.341954	6.040665
C	6.901484	31.150513	3.457193
H	5.943253	31.368355	3.927538
H	12.818552	29.947677	−2.313518
H	10.275149	30.309028	−3.219497
H	15.067008	29.716443	2.383386
H	14.183273	29.922724	4.947789
H	9.566157	30.810931	7.190820
H	7.040051	31.262492	6.288255
H	4.784041	31.462647	1.591602
H	5.645237	31.157910	−0.969763
O	11.271004	27.189015	0.241073
N	10.314956	27.092229	−0.492508
C	10.665599	26.630816	−1.839659
H	11.725605	26.360244	−1.903940
H	9.999662	25.796465	−2.089689
H	10.425978	27.467645	−2.509992
H	8.869302	28.341138	1.410069
H	10.346146	28.058503	1.721884
O	9.543553	28.476883	2.095882

=== TS1c ===

H	9.169411	32.978283	2.809201
S	9.931415	32.762003	1.712865
Fe	10.581461	30.564446	2.263698
N	11.619419	30.652308	0.548615
N	12.138891	31.340346	3.259909
N	9.473106	30.658410	3.923301
N	9.005003	29.833211	1.243898
C	11.180619	30.332011	−0.705218
C	12.250954	30.466400	−1.651851
C	13.346242	30.857776	−0.951725
C	12.940115	30.979107	0.419662
C	13.380732	31.588604	2.755170
C	14.230975	32.127100	3.782450
C	13.476661	32.216813	4.906188
C	12.170732	31.718976	4.568414
C	9.870942	31.108772	5.150251
C	8.820306	30.917866	6.109987
C	7.795200	30.316376	5.453731
C	8.213840	30.156594	4.089540
C	7.789223	29.483085	1.768704
C	6.911193	29.042947	0.723139
C	7.593564	29.171657	−0.445178
C	8.895883	29.671434	−0.113960
C	9.906828	29.898725	−1.030420
H	9.693130	29.698641	−2.079056
C	13.772226	31.400292	1.442176
H	14.803139	31.639029	1.185047
C	11.115232	31.630060	5.457207
H	11.285015	31.962809	6.480000
C	7.426572	29.593947	3.099220
H	6.428490	29.259821	3.379027
H	14.348950	31.060061	−1.313637
H	12.156158	30.269537	−2.714600
H	15.268542	32.407244	3.632938
H	13.757537	32.582232	5.888490
H	8.886499	31.202769	7.154883
H	6.830479	30.002114	5.838660
H	5.892924	28.702681	0.881141
H	7.262006	28.950225	−1.454320
O	11.918691	27.225952	1.048042
N	10.627992	27.165260	1.471057
C	10.349463	26.339378	2.606982
H	11.041169	26.571816	3.428711
H	9.324070	26.550192	2.930182
H	10.443593	25.277422	2.340555
H	10.038238	27.951295	1.203128
H	12.258473	28.011049	1.554220
O	11.186433	29.010587	2.664608

=== INT1c ===

H	8.863373	32.457306	1.061011
S	9.928448	32.719236	1.858138
Fe	10.539445	30.621424	2.284188
N	11.638108	30.50885	0.597742
N	12.109822	31.31055	3.292417
N	9.477908	30.567095	3.975947
N	8.984053	29.846562	1.266205
C	11.188623	30.267625	-0.677487
C	12.255216	30.433646	-1.621618
C	13.356869	30.799211	-0.908438
C	12.953991	30.872921	0.466491
C	13.365607	31.528266	2.803915
C	14.227891	32.046653	3.837087
C	13.471263	32.14444	4.958503
C	12.155529	31.671866	4.606976
C	9.860871	31.032981	5.199951
C	8.793057	30.882914	6.152106
C	7.754744	30.316228	5.483726
C	8.191066	30.137286	4.123888
C	7.744713	29.540936	1.776251
C	6.854616	29.139212	0.723111
C	7.553229	29.235474	-0.441302
C	8.873551	29.680968	-0.097018
C	9.89878	29.88102	-1.008456
H	9.677283	29.712481	-2.06211
C	13.771833	31.309807	1.497731
H	14.805858	31.547988	1.248235
C	11.104697	31.562643	5.502839
H	11.275907	31.896078	6.52616
C	7.384256	29.63924	3.111763
H	6.36668	29.351974	3.377228
H	14.355065	31.024348	-1.27129
H	12.158418	30.288973	-2.693286
H	15.274177	32.299948	3.696029
H	13.757888	32.491545	5.946404
H	8.850126	31.179936	7.194762
H	6.769903	30.050029	5.855564
H	5.8214	28.83953	0.870801
H	7.218518	29.025396	-1.452606
O	11.771179	27.445651	0.994929
N	10.815967	27.754753	2.024998
C	10.607346	26.487912	2.754326
H	11.54836	26.267673	3.262665
H	9.806576	26.664089	3.47686
H	10.346503	25.704287	2.038198
H	9.955256	28.047034	1.518549
H	12.106768	28.341533	0.768977
O	11.216996	28.732144	2.813965

=== RCd ===

H	0.828943	2.768958	−0.654897
S	−0.388326	2.607601	−0.088973
Fe	−0.294384	0.126760	0.059375
N	0.695771	0.021223	−1.677516
N	1.430562	0.529942	0.996877
N	−1.290956	0.500857	1.757496
N	−2.031745	0.000329	−0.921921
C	0.163438	−0.294824	−2.898928
C	1.197350	−0.395835	−3.887684
C	2.368158	−0.125924	−3.253438
C	2.037738	0.125083	−1.879527
C	2.688025	0.568454	0.452593
C	3.676255	0.762130	1.476407
C	3.008672	0.811920	2.654207
C	1.613135	0.667346	2.345410
C	−0.752808	0.646971	3.002752
C	−1.789545	0.784975	3.987822
C	−2.968489	0.729897	3.320064
C	−2.645101	0.537275	1.933229
C	−3.285830	0.094587	−0.398252
C	−4.276682	−0.150857	−1.407288
C	−3.600539	−0.410599	−2.555584
C	−2.204522	−0.306260	−2.237765
C	−1.183255	−0.461670	−3.162322
H	−1.466430	−0.710228	−4.184578
C	2.976008	0.397907	−0.887654
H	4.021684	0.458789	−1.189256
C	0.601367	0.696593	3.286726
H	0.891633	0.804638	4.330861
C	−3.580154	0.369153	0.929018
H	−4.631782	0.419780	1.208738
H	3.373485	−0.096427	−3.660590
H	1.026917	−0.632530	−4.932725
H	4.743479	0.829409	1.294206
H	3.405357	0.935684	3.656138
H	−1.612689	0.917639	5.049932
H	−3.977618	0.801156	3.711934
H	−5.347728	−0.128134	−1.236001
H	−3.992513	−0.643958	−3.539915
O	2.244857	−2.579894	1.061585
N	2.858071	−2.822707	−0.172734
C	1.919117	−3.398554	−1.111796
H	1.009384	−2.781496	−1.253119

H	2.425586	−3.523530	−2.077823
H	1.610864	−4.384078	−0.743160
H	3.183951	−1.915495	−0.522771
H	1.345661	−2.204200	0.876019
O	−0.216510	−1.484070	0.363210

=== TS1d ===

H	11.308919	32.985704	1.754247
S	9.994187	32.739316	1.950687
Fe	10.255965	30.401695	2.295530
N	11.253827	30.265996	0.568850
N	11.981579	30.787273	3.240589
N	9.246952	30.657804	4.015257
N	8.511431	30.208713	1.317469
C	10.709756	30.035009	−0.669036
C	11.744444	29.983684	−1.660617
C	12.919860	30.204132	−1.015354
C	12.601238	30.386517	0.371849
C	13.236373	30.823921	2.700963
C	14.212473	31.073379	3.723747
C	13.532612	31.182463	4.892651
C	12.142272	31.004137	4.583123
C	9.784091	30.888361	5.245949
C	8.744957	30.963183	6.236515
C	7.570700	30.783607	5.582284
C	7.896365	30.595551	4.194694
C	7.259725	30.215403	1.859532
C	6.275122	29.995264	0.837170
C	6.951552	29.848596	−0.330017
C	8.346878	29.984208	−0.016253
C	9.365037	29.891246	−0.949268
H	9.084505	29.707787	−1.985389
C	13.540218	30.634908	1.362516
H	14.584923	30.712736	1.063043
C	11.130655	31.047240	5.522580
H	11.413912	31.228705	6.558180
C	6.960010	30.396842	3.197821
H	5.910389	30.377931	3.487010
H	13.923275	30.254500	−1.425871
H	11.569938	29.813782	−2.718126
H	15.280576	31.146601	3.547193
H	13.918574	31.365590	5.889927
H	8.918351	31.138180	7.293218
H	6.561479	30.776219	5.980650
H	5.205405	29.958736	1.015065
H	6.561377	29.667084	−1.326081
O	12.694924	27.692771	3.484245
N	12.955470	27.472508	2.152586
C	11.829246	27.097803	1.417567
H	10.945118	27.908858	1.712079
H	12.026571	27.099337	0.341311
H	11.403364	26.154237	1.778697
H	13.487594	28.261504	1.773105
H	11.791252	28.124540	3.494345
O	10.294864	28.693607	2.652920

=== PROd ===

H	−0.273125	2.613750	−1.601667
S	−0.384427	2.474871	−0.258332
Fe	−0.295248	0.255344	−0.190342
N	0.569211	0.102585	−1.992092
N	1.517608	0.289040	0.677541
N	−1.142003	0.280419	1.616543
N	−2.086100	0.087196	−1.052759
C	−0.052788	0.025322	−3.209014
C	0.914472	−0.001327	−4.272323
C	2.136679	0.068575	−3.687977
C	1.912818	0.141105	−2.269422
C	2.727794	0.340280	0.040677
C	3.787719	0.494872	0.991329
C	3.201631	0.561178	2.223317
C	1.787149	0.448221	2.010698
C	−0.522333	0.389687	2.831499
C	−1.491547	0.420663	3.894765
C	−2.709747	0.337341	3.307938
C	−2.478767	0.257074	1.888970
C	−3.296200	0.099863	−0.421118
C	−4.364868	0.024095	−1.380784
C	−3.784544	−0.028664	−2.605286
C	−2.363535	0.013782	−2.387859
C	−1.423299	−0.018643	−3.402795
H	−1.787418	−0.077031	−4.427788
C	2.921547	0.244144	−1.330489
H	3.947354	0.283371	−1.696295
C	0.842247	0.472152	3.025757
H	1.203146	0.577702	4.048133
C	−3.489390	0.170620	0.946371
H	−4.516436	0.165749	1.309374
H	3.115790	0.078881	−4.156407
H	0.665945	−0.064016	−5.326914
H	4.840873	0.564676	0.736372
H	3.669991	0.703119	3.192641
H	−1.244806	0.495772	4.948778
H	−3.691115	0.329604	3.771109

H	−5.419473	0.013812	−1.125038
H	−4.254775	−0.093837	−3.581203
O	1.210331	−2.875426	1.915096
N	2.372286	−2.567163	1.511394
C	3.099117	−3.205389	0.651345
H	0.249169	−2.135960	−0.865315
H	4.073192	−2.799432	0.402750
H	2.705445	−4.121877	0.223124
H	2.769434	−1.691270	1.903478
H	0.119878	−2.224364	0.696181
O	−0.312021	−1.869512	−0.121506

=== 1[Fe(II)(Por)(SH)] ===

H	0.335381	3.171983	−0.275428
S	−0.840205	2.524531	−0.081072
Fe	−0.028724	0.397057	−0.193407
N	0.906092	0.690036	−1.929241
N	1.663053	0.825888	0.770731
N	−0.871485	−0.149665	1.530284
N	−1.630837	−0.279501	−1.171169
C	0.369822	0.580836	−3.184021
C	1.324464	0.969112	−4.188673
C	2.455186	1.326610	−3.528524
C	2.179781	1.155145	−2.126306
C	2.842887	1.271181	0.234265
C	3.802543	1.558781	1.267076
C	3.188742	1.291062	2.448085
C	1.860501	0.840913	2.125838
C	−0.347722	−0.007613	2.786775
C	−1.292872	−0.423594	3.789713
C	−2.410975	−0.813525	3.126951
C	−2.138678	−0.631030	1.725269
C	−2.802093	−0.744843	−0.635180
C	−3.756656	−1.048894	−1.668408
C	−3.154941	−0.751008	−2.847924
C	−1.838196	−0.268270	−2.524431
C	−0.912809	0.144609	−3.466900
H	−1.211909	0.108654	−4.515646
C	3.087795	1.431905	−1.118938
H	4.071481	1.801001	−1.413792
C	0.921810	0.464015	3.070880
H	1.211472	0.527995	4.120963
C	−3.046792	−0.905574	0.717678
H	−4.028217	−1.280850	1.011992
H	3.400359	1.680336	−3.931544
H	1.129689	0.960457	−5.257675
H	4.811127	1.920971	1.086913
H	3.579975	1.382612	3.457714
H	−1.101135	−0.408888	4.859181
H	−3.347605	−1.191415	3.527925
H	−4.754626	−1.439668	−1.489123
H	−3.547170	−0.841261	−3.857255

=== 5[Fe(II)(Por)(SH)] ===

H	9.634138	33.876919	1.092715
S	10.194736	33.656709	2.307464
Fe	10.327623	31.349092	1.785721
N	10.837970	31.243188	−0.300012
N	12.331492	30.623609	2.103722
N	9.862046	30.194253	3.537351
N	8.359033	30.795440	1.130223
C	9.971219	31.447431	−1.328401
C	10.702178	31.709517	−2.547146
C	12.023902	31.652014	−2.226544
C	12.091494	31.357095	−0.814003
C	13.378090	30.829905	1.258933
C	14.625698	30.593406	1.948785
C	14.303447	30.236482	3.221859
C	12.861137	30.258440	3.301855
C	10.727694	29.884642	4.539500
C	9.997167	29.477440	5.717572
C	8.676186	29.547373	5.397428
C	8.608063	29.994417	4.025164
C	7.313965	30.515393	1.953682
C	6.069466	30.615092	1.226146
C	6.393275	30.960472	−0.050031
C	7.833665	31.068700	−0.093521
C	8.579301	31.386309	−1.230723
H	8.016565	31.581595	−2.145905
C	13.270600	31.184352	−0.087033
H	14.206781	31.313395	−0.633838
C	12.119263	29.929800	4.437825
H	12.685440	29.657380	5.330807
C	7.427073	30.160377	3.298995
H	6.494773	29.964619	3.832605
H	12.882761	31.787562	−2.878649
H	10.250073	31.900122	−3.517117
H	15.612208	30.685494	1.501777
H	14.970016	29.973180	4.038981
H	10.449017	29.172142	6.657824
H	7.817276	29.310725	6.020000
H	5.083775	30.438482	1.648818
H	5.728137	31.125384	−0.893687

=== 2[Fe(III)(Por)(SH)] ===

H	−0.276027	2.983643	−1.165396
S	−0.781354	2.503168	−0.003303
Fe	−0.018729	0.432916	−0.172860
N	0.907686	0.744312	−1.904930
N	1.700218	0.729289	0.781741
N	−0.864824	−0.107827	1.547845
N	−1.566384	−0.335769	−1.156788
C	0.378584	0.635989	−3.166465
C	1.320099	1.073395	−4.159096
C	2.439732	1.446701	−3.492111
C	2.175404	1.234004	−2.096386
C	2.867003	1.224053	0.250123
C	3.820473	1.502046	1.281548
C	3.216160	1.203944	2.460623
C	1.897068	0.746811	2.141834
C	−0.338107	0.006633	2.808280
C	−1.298240	−0.379515	3.805299
C	−2.422837	−0.737835	3.139633
C	−2.141332	−0.569515	1.740539
C	−2.757111	−0.762270	−0.619441
C	−3.706175	−1.056373	−1.650446
C	−3.098408	−0.773721	−2.831559
C	−1.782403	−0.307348	−2.513968
C	−0.878560	0.143858	−3.456800
H	−1.181743	0.112682	−4.502663
C	3.094923	1.461728	−1.091856
H	4.069434	1.856633	−1.376749
C	0.952289	0.407213	3.091897
H	1.247378	0.462827	4.139015
C	−3.031909	−0.872456	0.730450
H	−4.021439	−1.227115	1.016364
H	3.377130	1.823318	−3.888519
H	1.128991	1.073947	−5.227286
H	4.818982	1.889237	1.106410
H	3.608724	1.291643	3.468473
H	−1.107362	−0.378098	4.873489
H	−3.365988	−1.099003	3.536606
H	−4.711408	−1.424208	−1.472196
H	−3.494232	−0.857097	−3.838506

=== 6[Fe(III)(Por)(SH)] ===

H	−1.228126	2.893053	−1.338382
S	−0.871030	2.919251	−0.032138
Fe	−0.111271	0.765131	−0.143789
N	0.953563	0.739126	−1.933497
N	1.728578	0.842411	0.829375
N	−0.854231	−0.118819	1.586321
N	−1.627265	−0.222257	−1.176970
C	0.430388	0.588952	−3.190105
C	1.404574	0.962907	−4.177700
C	2.522478	1.343804	−3.502890
C	2.232609	1.200049	−2.103350
C	2.901570	1.287410	0.280752
C	3.872481	1.518010	1.314203
C	3.272812	1.205163	2.494085
C	1.934239	0.786230	2.181433
C	−0.301657	−0.049844	2.836882
C	−1.251943	−0.489088	3.820745
C	−2.387376	−0.821452	3.149894
C	−2.129707	−0.588850	1.756020
C	−2.797238	−0.676845	−0.628129
C	−3.736668	−0.996936	−1.666612
C	−3.120676	−0.731960	−2.849996
C	−1.805498	−0.247014	−2.534443
C	−0.854143	0.139057	−3.471133
H	−1.136646	0.071460	−4.521427
C	3.130612	1.467754	−1.077600
H	4.116409	1.830247	−1.368132
C	0.990544	0.378021	3.116148
H	1.294526	0.384688	4.162518
C	−3.036027	−0.831693	0.731684
H	−4.018455	−1.204123	1.020954
H	3.469899	1.688301	−3.904846
H	1.241378	0.928579	−5.250115
H	4.884967	1.869311	1.143267
H	3.689344	1.247115	3.495353
H	−1.059058	−0.535698	4.887688
H	−3.322364	−1.200215	3.550116
H	−4.736037	−1.384892	−1.497627
H	−3.509848	−0.854504	−3.855713

=== nitrosomethane ===

O	1.907675	−1.738848	−0.531995
N	0.740258	−1.491717	−0.374691
C	−0.079648	−2.701952	−0.142405
H	−0.578649	−2.569314	0.825799
H	−0.860886	−2.714637	−0.912650
H	0.534959	−3.609287	−0.165361

=== 1MIC(II) ===

H	0.307069	3.082113	−0.312996
S	−0.845960	2.414204	−0.068202
Fe	0.045218	0.224998	−0.204900

N	0.923450	0.682880	−1.943412
N	1.686658	0.815086	0.776304
N	−0.864784	−0.196041	1.542321
N	−1.631280	−0.320354	−1.178725
C	0.391753	0.541064	−3.188655
C	1.331441	0.973117	−4.193756
C	2.441968	1.382277	−3.530806
C	2.172837	1.191311	−2.127037
C	2.837181	1.305734	0.238001
C	3.792031	1.611953	1.274121
C	3.198278	1.291002	2.450823
C	1.883522	0.796606	2.123440
C	−0.325940	−0.070514	2.788090
C	−1.290068	−0.432153	3.795948
C	−2.431669	−0.759951	3.138356
C	−2.153463	−0.601602	1.733083
C	−2.819379	−0.712322	−0.634827
C	−3.780245	−0.996816	−1.670173
C	−3.154590	−0.760978	−2.852110
C	−1.819264	−0.326500	−2.529171
C	−0.876350	0.055991	−3.471743
H	−1.168404	0.002208	−4.521554
C	3.071442	1.492335	−1.115353
H	4.040580	1.898855	−1.407762
C	0.958984	0.371208	3.065685
H	1.255522	0.418493	4.114549
C	−3.073284	−0.843260	0.722792
H	−4.073549	−1.162210	1.018968
H	3.369980	1.780703	−3.931424
H	1.138554	0.959840	−5.262910
H	4.782835	2.021387	1.097738
H	3.591031	1.376776	3.460219
H	−1.098244	−0.415797	4.865264
H	−3.388318	−1.075661	3.545356
H	−4.800262	−1.325239	−1.490555
H	−3.546768	−0.850653	−3.861412
O	1.916914	−1.757958	−0.594499
N	0.749436	−1.489213	−0.326920
C	−0.093047	−2.691076	−0.126953
H	−0.620764	−2.593767	0.828277
H	−0.839826	−2.717518	−0.930224
H	0.550996	−3.576224	−0.150982

=== 2MIC(III) ===

H	−1.094924	2.556982	−1.288388
S	−0.695740	2.420141	−0.001869
Fe	0.052874	0.314442	−0.180894

N	0.953753	0.712780	−1.911743
N	1.717902	0.801446	0.803622
N	−0.832696	−0.158471	1.553565
N	−1.594903	−0.248527	−1.164344
C	0.451585	0.521125	−3.169380
C	1.393245	0.968537	−4.157973
C	2.466190	1.450527	−3.481557
C	2.185448	1.278967	−2.082768
C	2.847681	1.357118	0.274518
C	3.810129	1.614950	1.310398
C	3.252266	1.197932	2.474459
C	1.942436	0.704403	2.148207
C	−0.272038	−0.125212	2.800855
C	−1.244795	−0.478311	3.796189
C	−2.412049	−0.700081	3.138827
C	−2.145220	−0.499332	1.741637
C	−2.805369	−0.585429	−0.619491
C	−3.750957	−0.884928	−1.657229
C	−3.099647	−0.728978	−2.839500
C	−1.762380	−0.316305	−2.521774
C	−0.801654	0.011722	−3.464327
H	−1.072387	−0.085192	−4.514966
C	3.068141	1.604777	−1.068220
H	4.019990	2.052277	−1.350759
C	1.032637	0.243091	3.083902
H	1.346458	0.225243	4.126747
C	−3.076090	−0.687891	0.734697
H	−4.087542	−0.966967	1.027068
H	3.382560	1.879094	−3.874429
H	1.228376	0.918404	−5.229374
H	4.788029	2.054857	1.143150
H	3.666450	1.223584	3.477109
H	−1.042416	−0.527967	4.861167
H	−3.379286	−0.978561	3.544763
H	−4.781175	−1.177730	−1.481401
H	−3.479175	−0.860677	−3.847650
O	1.907318	−1.815200	−0.584335
N	0.758580	−1.565657	−0.316978
C	−0.108752	−2.737310	−0.106441
H	−0.584438	−2.623444	0.875037
H	−0.897398	−2.697314	−0.868421
H	0.491202	−3.648582	−0.179433

=== 1MIC(II), O-bound ===

H	−0.476022	2.756138	1.103542
S	−0.758689	2.405175	−0.174467
Fe	0.109775	0.261537	−0.078637

N	1.008754	0.532989	−1.844076	N	1.033342	0.608432	−1.880073
N	1.751460	0.931308	0.873062	N	1.742989	0.971322	0.830242
N	−0.768490	−0.058016	1.703408	N	−0.712255	−0.162395	1.616492
N	−1.508577	−0.449302	−1.015724	N	−1.476168	−0.408223	−1.090673
C	0.493816	0.280092	−3.080556	C	0.518637	0.390526	−3.129734
C	1.437727	0.640763	−4.107922	C	1.439388	0.831243	−4.137845
C	2.534097	1.123295	−3.469839	C	2.513291	1.345314	−3.484978
C	2.250627	1.051846	−2.058486	C	2.246138	1.209533	−2.081716
C	2.895046	1.396220	0.294683	C	2.867714	1.514235	0.270755
C	3.839778	1.812707	1.300392	C	3.797001	1.916721	1.290942
C	3.246031	1.592280	2.501149	C	3.226847	1.604847	2.481437
C	1.942999	1.044392	2.217735	C	1.948486	1.019813	2.183112
C	−0.247420	0.181846	2.939820	C	−0.191301	0.039293	2.867336
C	−1.199988	−0.157244	3.967413	C	−1.137083	−0.345675	3.874542
C	−2.311936	−0.602735	3.328745	C	−2.254078	−0.758539	3.221298
C	−2.027816	−0.533652	1.917271	C	−1.982818	−0.632236	1.818641
C	−2.669512	−0.868936	−0.437933	C	−2.644627	−0.848605	−0.531655
C	−3.617609	−1.278427	−1.443199	C	−3.580371	−1.233578	−1.552731
C	−3.009906	−1.096244	−2.643104	C	−2.964594	−1.024675	−2.743036
C	−1.696502	−0.575384	−2.359982	C	−1.658231	−0.505265	−2.443653
C	−0.767348	−0.237465	−3.331433	C	−0.728012	−0.145252	−3.402117
H	−1.053520	−0.382564	−4.374180	H	−1.008342	−0.263451	−4.448189
C	3.134314	1.456942	−1.069608	C	3.106906	1.635938	−1.086055
H	4.097027	1.856441	−1.392584	H	4.045671	2.094854	−1.394317
C	1.017908	0.692409	3.189853	C	1.055112	0.574929	3.140969
H	1.303849	0.838877	4.232657	H	1.341389	0.677831	4.187080
C	−2.919469	−0.912178	0.925281	C	−2.891601	−0.948953	0.825042
H	−3.898543	−1.273827	1.243590	H	−3.869934	−1.314004	1.135533
H	3.460643	1.501766	−3.893156	H	3.415187	1.785873	−3.897977
H	1.258588	0.534129	−5.174351	H	1.261507	0.758505	−5.205970
H	4.824547	2.221248	1.090158	H	4.762685	2.372596	1.096700
H	3.633425	1.778032	3.499312	H	3.618453	1.747929	3.483509
H	−1.019196	−0.054121	5.034028	H	−0.953938	−0.288038	4.942746
H	−3.250374	−0.950731	3.751940	H	−3.191294	−1.117881	3.634338
H	−4.615920	−1.652454	−1.232587	H	−4.576675	−1.618176	−1.359198
H	−3.397281	−1.283257	−3.640903	H	−3.342953	−1.194909	−3.745910
O	0.845231	−1.504131	0.063264	O	1.053747	−1.622869	−0.201512
N	0.847131	−2.326165	−0.864627	N	0.476148	−2.606413	0.204371
C	1.473915	−3.579331	−0.445032	C	1.264301	−3.827586	0.043051
H	0.735633	−4.386216	−0.559191	H	1.363828	−4.274024	1.040618
H	2.303922	−3.795676	−1.132668	H	0.656775	−4.515159	−0.559161
H	1.831157	−3.525118	0.591988	H	2.238378	−3.628138	−0.417725

=== 2MIC(III), O-bound ===

H	−0.059808	2.902287	0.830861
S	−0.814011	2.352794	−0.151626
Fe	0.107452	0.336487	−0.128714
